# Giant cell tumor of soft tissue of the colon: a case report and review of the literature

**DOI:** 10.1186/s12876-022-02391-x

**Published:** 2022-06-27

**Authors:** Seung Woo Lee, Jun Lee, Seong Jung Kim, Ran Hong

**Affiliations:** 1 Department of Internal Medicine, College of Medicine, Chosun University, 309, Pilmun-daero, Dong-gu, Gwangju, 61452 Republic of Korea; 2 Department of Pathology, College of Medicine, Chosun University, Gwangju, Republic of Korea

**Keywords:** Giant cell tumors, Soft tissue neoplasm, Endoscopic mucosal resection, Case report

## Abstract

**Background:**

A giant cell tumor (GCT) is a benign neoplasm characterized by mixture of mononuclear cells and multinucleated cells. A GCT of soft tissue (GCT-ST) is developed in various extraosseous sites, but GCT-ST of the gastrointestinal tract is very rare. GCT-ST usually has a benign course, but rare cases reported malignant potential of the tumor. Therefore, complete resection is required to prevent local recurrence or distant metastasis.

**Case presentation:**

A 53-year-old woman was admitted for follow-up colonoscopy who underwent the colorectal endoscopic submucosal dissection (ESD) of a laterally spreading tumor at the hepatic flexure 6 months ago. A colonoscopy showed a polypoid mass about 3.5 × 2.5 cm at the previous ESD site. As endoscopic finding showed a smooth multi-nodular tumor without submucosal invasion, we performed endoscopic mucosal resection. Based on pathological and immunohistochemical findings, the lesion was diagnosed as a GCT-ST in the colon. Follow-up colonoscopy performed 6 months later revealed no evidence of recurrence.

**Conclusion:**

This is the first report of a GCT-ST identified in the colon. Although GCT-ST generally has a benign clinical course, complete resection should be performed to prevent local recurrence and metastasis.

## Background

A giant cell tumor (GCT) is a benign osteolytic skeletal neoplasm characterized by multinucleated osteoclast-like giant cells (OGCs) distributed between stromal cells. A GCT of soft tissue (GCT-ST) is a GCT that originates in a region other than the bone, most often in the lower extremities, followed by the trunk, upper extremities, and neck [1]. Although a GCT-ST has very similar clinical features and pathological findings to those of a GCT of the bone, its exact pathogenesis has not been fully elucidated. Most cases of GCT-ST have a benign course, but rare cases of local recurrence or distant metastases have been reported [2]. Therefore, complete excision is necessary whenever possible. GCT-ST of the gastrointestinal tract is very rare, and to the best of our knowledge, this is the first case of a case in the colon. Here we report a case of GCT-ST of the colon that was completely resected endoscopically as well as a review of the literature.

## Case presentation

**Fig. 1:**
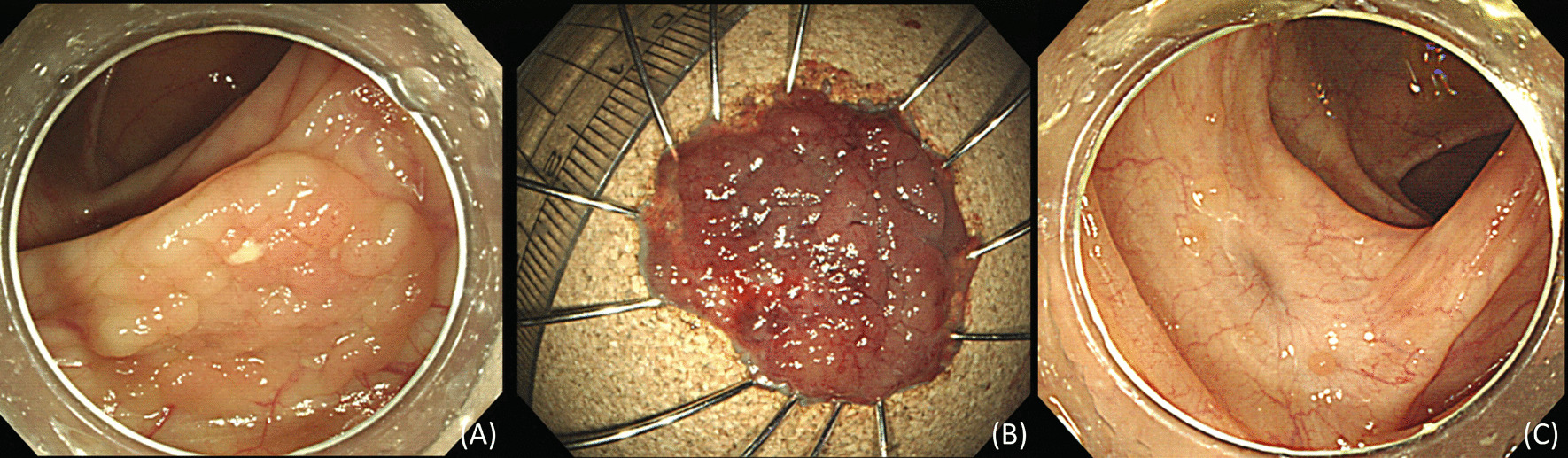
Initial colonoscopy. Colonoscopy image showing nodular mixed granular type of LST at the hepatic flexure (**A**). En-bloc resected tumor measuring 25 × 20 mm (**B**). Follow-up endoscopy performed after 3 months showing a fibrotic scar at the endoscopic submucosal dissection site (**C**)

A 53-year-old woman visited our hospital for follow-up colonoscopy after endoscopic resection. She had undergone breast-conserving surgery and chemoradiation therapy for right breast cancer 5 years ago. She was a non-smoker and had no history of alcohol consumption. She underwent the colorectal endoscopic submucosal dissection (ESD) of a 2.5 × 2 cm laterally spreading tumor at the hepatic flexure 6 months ago (Fig. 1). The lesion was completely removed without complications, and a histopathological examination confirmed the presence of a tubular adenoma with low-grade dysplasia.

**Fig. 2:**
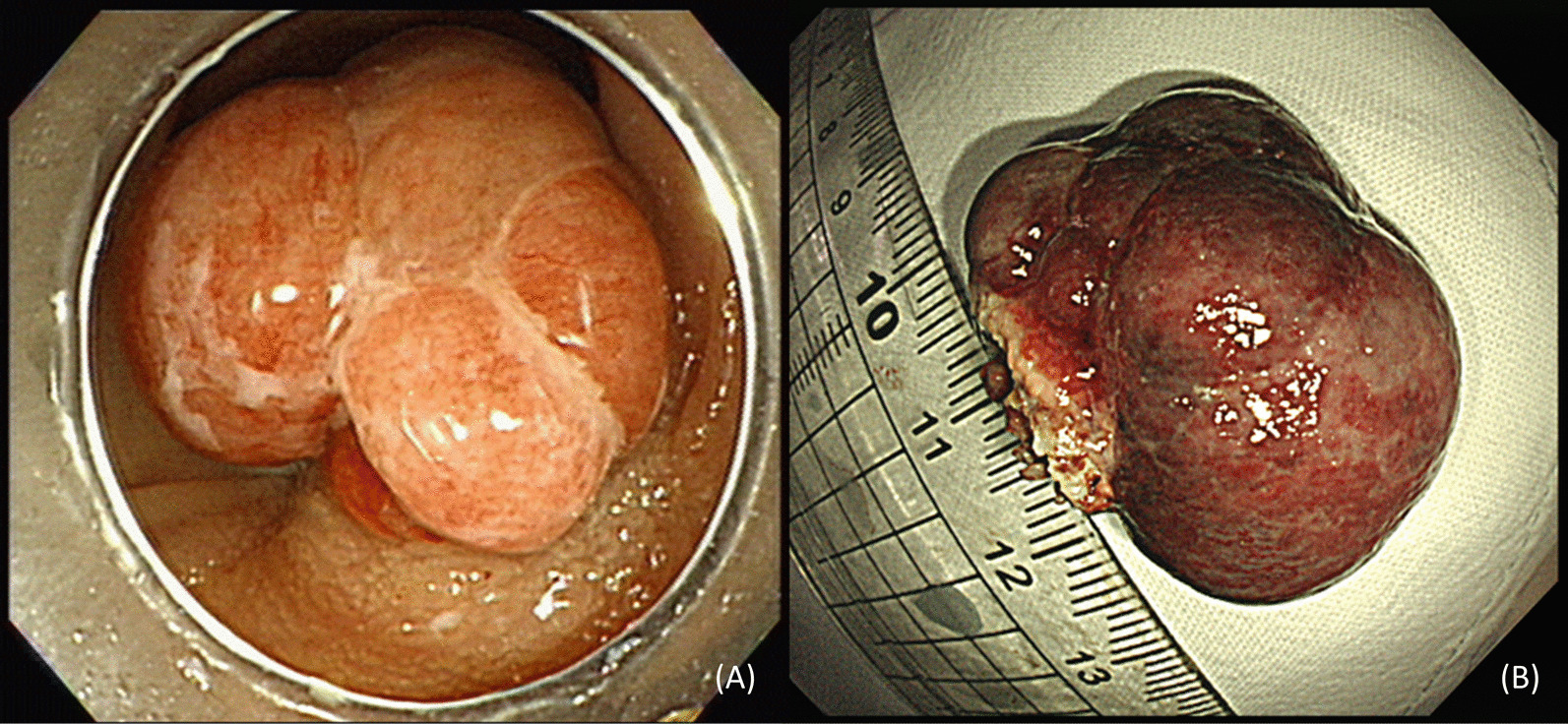
Follow-up colonoscopy. Colonoscopy image showing a circumscribed multi-nodular polypoid tumor (Paris classification Ip type) at the previous endoscopic submucosal dissection site (**A**). En-bloc resected specimen measuring 35 × 25 mm (**B**)

**Fig. 3:**
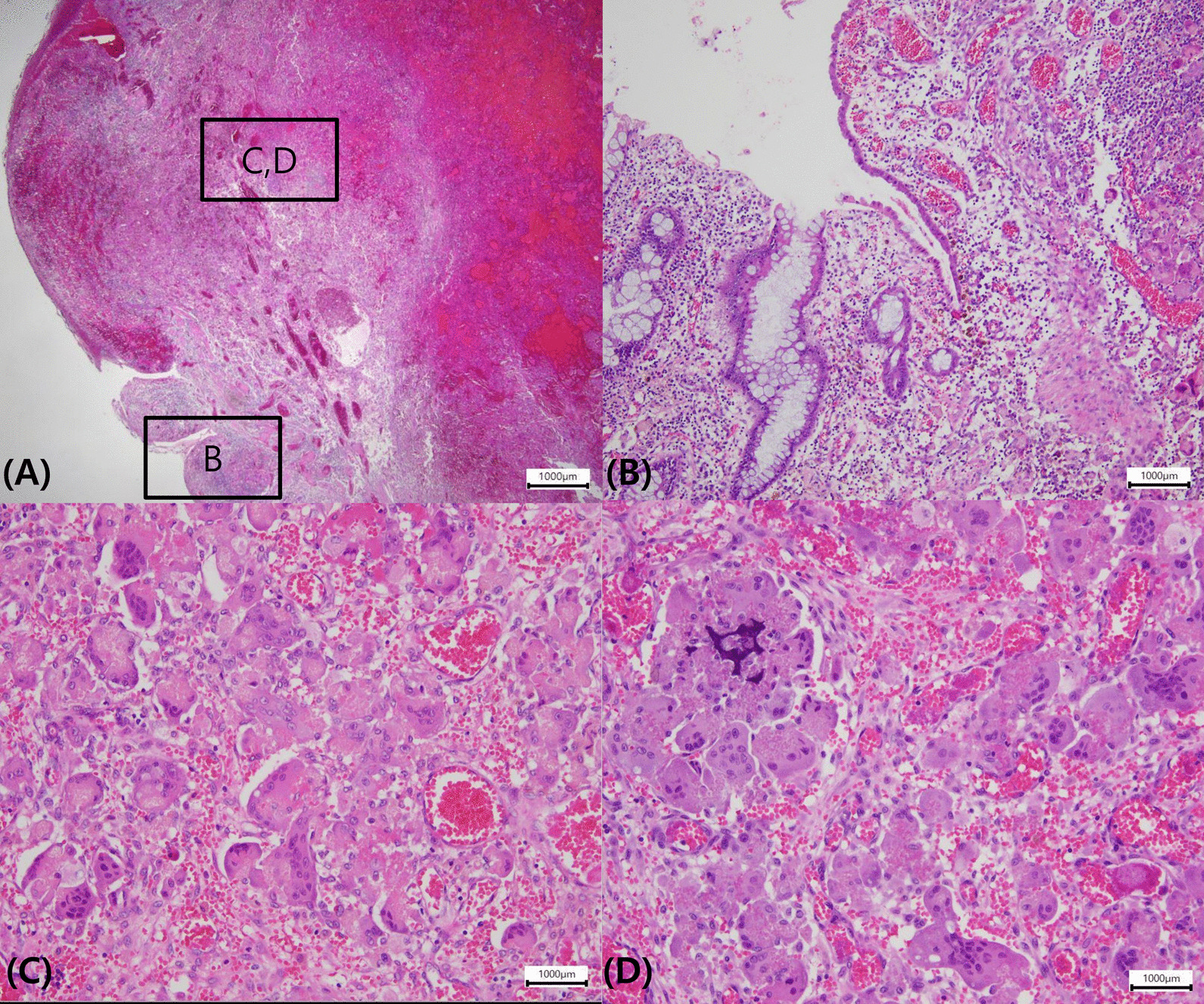
Histopathological findings of endoscopic mucosal resection specimen. A mixture of mononuclear cells and multinucleated osteoclast-like giant cells were localized in the center of the tumor with diffused internal hemorrhagic necrosis (**A**). Remnant hyperplastic crypts is observed in the basal portion (**B**). In the high power, proliferating multinucleated osteoclast-like giant cells and mononuclear cells with vascular proliferation are evident (**C**, **D**). (Hematoxylin and eosin stain: **A **× 1.25; **B** × 4; **C** × 20; **D** × 20) (microscope model: Olympus BX43F/software: KOPTIC HKBasic × 64, 4.8.16384.20200113, resolution: 600 dpi)

**Fig. 4:**
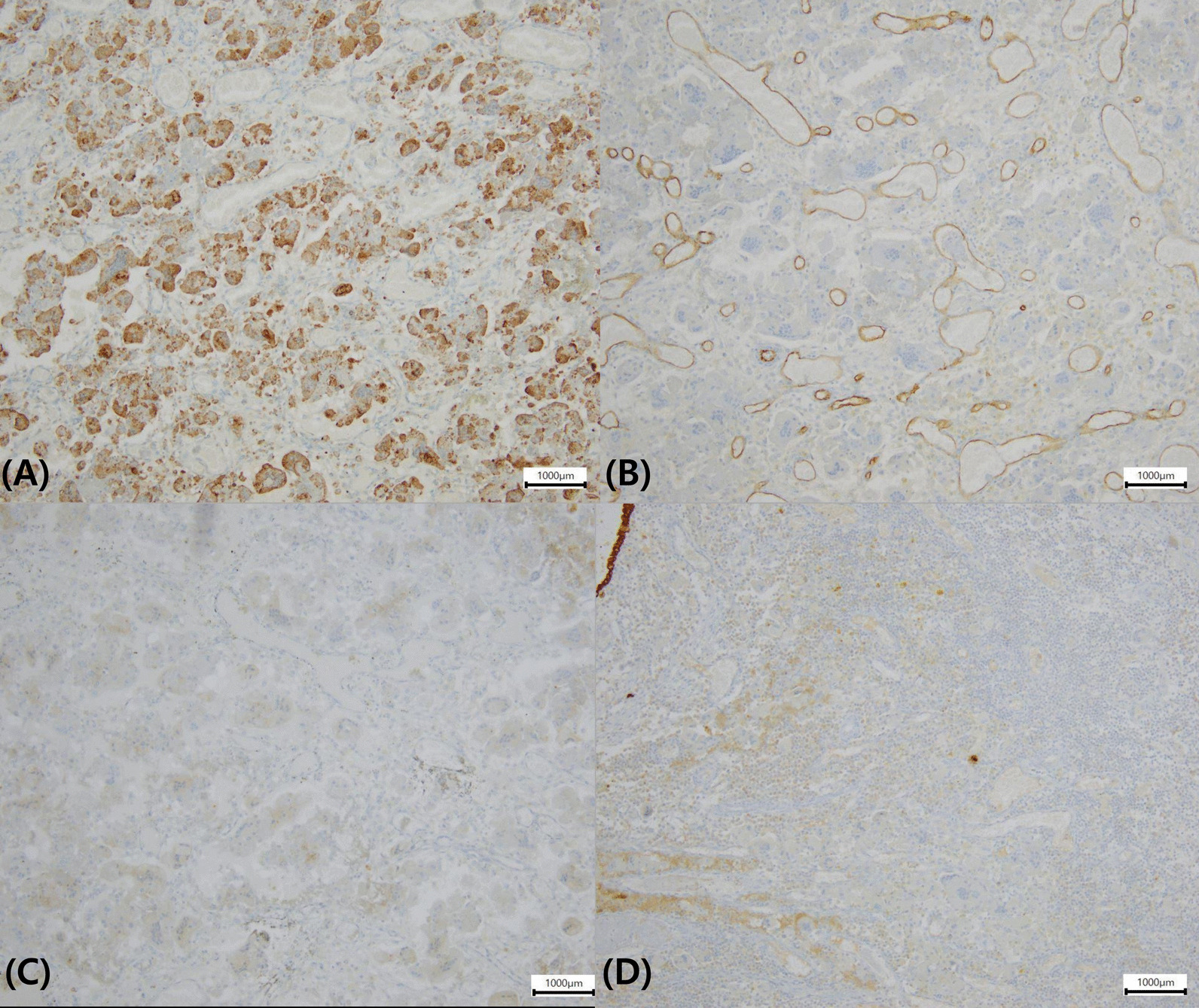
Immunohistochemical findings of the tumor. The tumor cells are immunoreactive for CD68 in the cytoplasm (**A**), but negative for CD34 (**B**), CD117 (**C**) and cytokeratin (**D**). (magnification: **A** × 100; **B** × 100; **C** × 100; **D** × 100) (microscope model: Olympus BX43F/software: KOPTIC HKBasic × 64, 4.8.16384.20200113, resolution: 600 dpi)

**Fig. 5:**
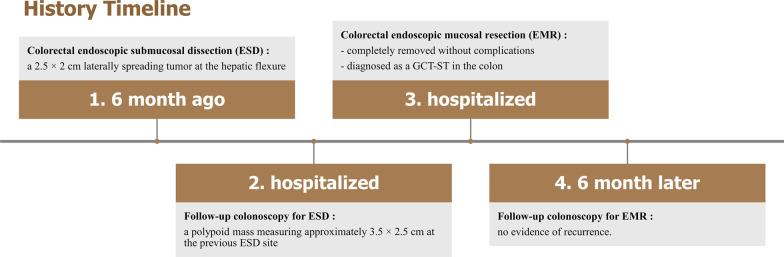
Timeline. History timeline of the patient with giant cell tumor of soft tissue of the colon

Follow-up colonoscopy showed a polypoid mass measuring approximately 3.5 × 2.5 cm at the previous ESD site. Endoscopic findings showed a smooth multi-nodular pedunculated tumor with focal erosion (Fig. 2). As there were no findings suspicious of invasive cancer, we performed endoscopic mucosal resection. A 0.9% saline solution mixed with indigo carmine and epinephrine was injected into the submucosa around the lesion to enable lifting, and the tumor was cut using an electrical snare. The mass was completely removed without complications within 8 min. Pathologically, the tumor was characterized by the proliferation of multinucleated OGCs and mononuclear cells with diffused internal hemorrhagic necrosis (Fig. 3). Immunohistochemically, mononuclear and multinuclear cells were immunoreactive for CD 68, but negative for CD34, CD117, and cytokeratin (Fig. 4). Based on these findings, the lesion was diagnosed as a GCT-ST in the colon. Follow-up colonoscopy with biopsy performed 6 months later showed no evidence of recurrence. At the time of writing, the patient has a satisfactory course without problems. The overall clinical course of the patient is as follows (Fig. 5).

## Discussion and conclusions

Since GCT-ST was first reported in 1972, approximately 70 cases have been reported from various sites [3, 4]. GCT-ST commonly occurs in middle-aged adults, but there is no significant difference in the incidence by sex [1]. Such tumors generally grow slowly, but they reportedly grow to various sizes ranging from 1 to 10 cm. A GCT-ST rarely causes obstruction-related symptoms and is usually asymptomatic and discovered incidentally. Cases of GCT-ST are usually diagnosed based on histological findings consisting of several well-separated nodules composed of a mixture of monocytes and multinucleated OGCs. A GCT-ST generally has mitotic activity with 1 to more than 30 mitotic figures per 10 high-power fields, but cellular atypia and pleomorphism are lacking. Although OGCs are important histological components of GCT-ST, they can also be found in other soft tissue tumors such as leiomyosarcoma and gastrointestinal stromal tumor (GIST) originating from the soft tissues [5]. However, unlike those in GCT-ST, OGCs found in other soft tissue tumors are observed in a locally infiltrated form in some areas. The difference about distribution of OGCs in tumor is a characteristic that distinguishes GCT-ST from other tumors. Immunohistochemical staining is helpful for the differential diagnosis of GCT-ST. These tissues are strongly positive for CD68 and negative for CD34, CD117, smooth muscle antibody, and Ki-67, findings that can be used to exclude GIST and leiomyosarcoma [6].

Although the pathogenesis of GCT-ST remains uncertain, OGCs that recruit lesions by RANKL expression are considered to play an important role [7]. Several authors suggested that a chronic inflammatory condition may be associated with OGC proliferation [8]. This can be explained by the large influx of inflammatory cells such as neutrophils or macrophages to the wound site, which changes the microenvironment and induces OGC aggregation [9]. Our case occurred at the ESD resection site, and we hypothesized that it may have occurred during the healing process of an ulcer created therein. In some case reports, there are reports of hyperplastic polyps occurring at the endoscopic resection site [10]. However, GCT-ST differs macroscopically from hyperplastic polyps in its smooth surface and multinodular polypoid mass. In case of having these features, a biopsy should be performed, and a diagnosis should be confirmed through pathological examination. It is important to distinguish a GCT-ST from a hyperplastic polyp since the latter do not become malignant, whereas the former rarely does but has the potential. In a series of case studies, only 1 in 16 surgically resected patients (6.2%) had local recurrence and pulmonary metastasis [1]. Episodes of distant metastasis and tumor-related death are extremely rare if GCT-ST is treated with complete resection. Therefore, endoscopists aim for complete resection and pathologic review.

Here we presented the first case of GCT-ST identified in the colon. GCT-ST is very rare and generally has a benign clinical course. Complete resection should be performed to prevent recurrence and metastasis to other organs. Histological analysis and immunohistochemical staining may be helpful in the diagnosis of and differentiation from other tumors.

## Data Availability

This is a case report of a single patient, to protect privacy and respect confidentiality; none of the raw data has been made available in any public repository. If you would like to access the raw data and obtain detailed information, please contact us at the email address (leejun@med.chosun.ac.kr). The original reports, laboratory studies, imaging studies and outpatient clinic records are retained as per normal procedure within the medical records of our institution.

## Abbreviations

CT: Computer tomography
ESD: Endoscopic submucosal dissection
GCT: Giant cell tumor
GCT-ST: Giant cell tumor of soft tissue
GIST: Gastrointestinal stromal tumor
OGC: Osteoclast-like giant cell
RANKL: Receptor activator of nuclear factor kappa-B ligands
